# Epidemiology of spinal pain in children: a study within the Danish National Birth Cohort

**DOI:** 10.1007/s00431-019-03326-7

**Published:** 2019-02-20

**Authors:** Anne Cathrine Joergensen, Lise Hestbaek, Per Kragh Andersen, Anne-Marie Nybo Andersen

**Affiliations:** 1 Section of Epidemiology, Department of Public Health, Faculty of Health and Medical Science, University of Copenhagen, Oster Farimagsgade 5, bd. 24, DK-1014 Copenhagen K, Denmark; 2 Department of Sport Science and Clinical Biomechanics, University of Southern Denmark, Campusvej 55, 5230 Odense, Denmark; 3 Section of Biostatistics, Department of Public Health, Faculty of Health and Medical Science, University of Copenhagen, Oster Farimagsgade 5, 1014 Copenhagen K, Denmark

**Keywords:** Spinal pain, Neck pain, Back pain, School children, Epidemiology, Socio-demographic risk factors

## Abstract

This study aims to describe the prevalence of spinal pain among Danish children, explore the differential nature of spinal pain, and investigate socio-demographic factors predisposing spinal pain. A descriptive study of 46,726 11–14-year-olds participating in the Danish National Birth Cohort was conducted. Self-reported spinal pain (neck, middle back, and low back pain) was registered and classified according to severity. Socioeconomic data on children and their parents were identified in Statistics Denmark registers. Associations between socio-demographic factors and aspects of spinal pain were estimated using multinomial logistic regression models. To account for sample selection, inverse probability weighting (IPW) was applied. Almost 10% boys and 14% girls reported severe spinal pain, whereas around 30% of all children reported moderate pain. Effect estimates indicated the risk to increase with increasing age. Further, children without biological full siblings, not living with both of their parents, or children living in less-educated or lower-income families were more likely to experience spinal pain. The study conclusions were essentially unaffected by IPW.

*Conclusion*: A considerable number of children suffer from spinal pain, and it is more common among children in more disadvantaged families. Etiology of spinal pain needs to be explored further with the aim of informing efficient and targeted prevention.

**Table:** 

**What is Known:** • * Childhood spinal pain may cause marked discomfort and impairment in children*’*s everyday life*, *and is suggested as important predictor of later-in-life spinal pain*. • *Methodological heterogeneity in previous studies and complexity of measuring pain make inferences at a broader level inadequate*.	
**What is New:** • *Prevalence of severe spinal pain in 11*–*14*-*year*-*olds was estimated to almost 10% for boys and 14% for girls, and children in more disadvantaged families were more likely to experience spinal pain*. • * The results seemed unaffected by sample selection*.

**Electronic supplementary material:**

The online version of this article (10.1007/s00431-019-03326-7) contains supplementary material, which is available to authorized users.

## Introduction

Spinal pain (i.e., neck and back pain) constitutes a public health concern worldwide [29]. Historically, spinal pain was primarily studied in the working age population, but it has become increasingly acknowledged that vulnerability to spinal pain develops and becomes apparent already in childhood [1, 9, 27]. Spinal pain has been framed “as a long-term or recurrent condition rather than a series of unrelated episodes” [9], and epidemiological studies have characterized a prior history of spinal pain as an important predictor of spinal pain later in life [16, 24, 49]. In addition, spinal pain in children may cause marked discomfort and impairment in children’s everyday life and cause long-term problems. Research suggests that children and adolescents reporting spinal pain experience increased healthcare utilization, absenteeism or impairment in school, and restrictions in physical activity [27, 30, 42]. Likewise, children with spinal pain commonly experience the co-existence of other health complaints, physically and mentally [9, 13, 45, 48]. Therefore, studying spinal pain etiology in its earliest onset may be of value, and likewise targeting primary prevention towards the young population rather than the working age population could be beneficial.

A growing body of evidence indicates spinal pain onset to be around age 10–12, to increase in prevalence with age, and to approach adult levels around age 18 [6, 9, 24, 31, 33]. However, spinal pain prevalence in the young population varies considerably across studies with lifetime prevalence estimates ranging between 4 and 74% [24, 27]. This wide discrepancy can be explained by methodological limitations and heterogeneity as well as the complexity of measuring pain [11, 24, 27, 33, 48], resulting in imprecision and inadequacy to synthesize findings and to make inferences at a broader level.

Familial and social factors are assumed to be of importance for childhood health and pain experience [13, 41]. In spinal pain research, a relationship has been indicated for risk factors such as parental socioeconomic status [17, 36], biological vulnerability [10, 15], and parental pain behavior [7, 47]; however, the evidence of risk factors predisposing to spinal pain is conflicting mainly due to methodological limitations [9].

Overall, findings from epidemiological studies of childhood spinal pain are ambiguous, and little is known about the etiology of spinal pain, early life predictors, and specific influence of timing and duration of spinal pain episodes [1, 9, 16, 24, 49]. Therefore, we aimed to describe the epidemiology of spinal pain in 11–14-year-olds in the Danish National Birth Cohort (DNBC) and to explore the differential nature of spinal pain. Further, we aimed to provide a population-based prevalence estimate of spinal pain in Danish children using inverse probability weighting (IPW).

## Methods

### Study population

**Fig. 1:**
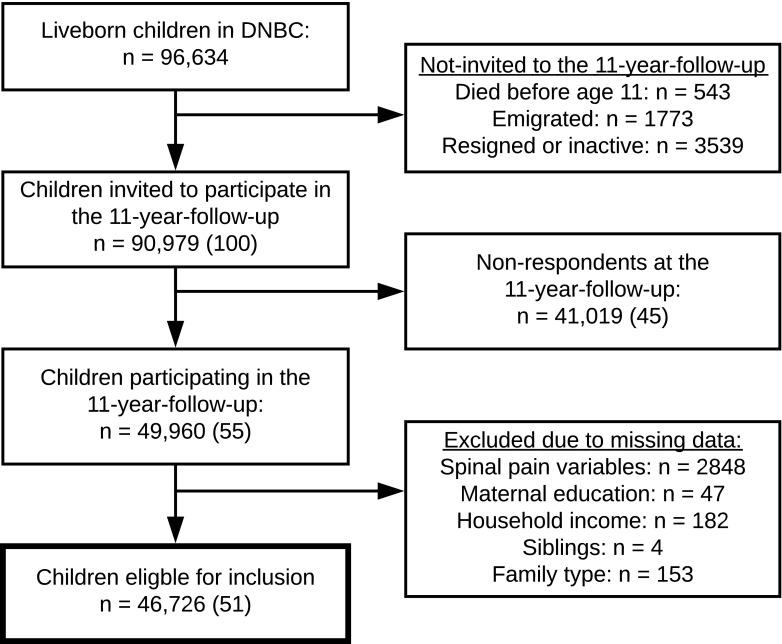
The flow chart for the selection of study participants eligible for inclusion in the study

For this descriptive cross-sectional study, we studied a cohort of 46,726 children born in Denmark from 1996 through 2003 participating in DNBC. DNBC is a population-based birth cohort of mothers and their children with several follow-ups going from pregnancy and through childhood and young adulthood [37]. Pregnant women (*n* = 100,415) were recruited during the period 1996 to 2002 by their general practitioner at their first antenatal visit around gestational weeks 6–12. Further details of DNBC are described elsewhere [37]. For this study, we used data from the 11-year follow-up (DNBC-11) for which children received an electronic questionnaire around their 11th birthday. Due to financial delay, DNBC-11 was carried out from 2010 to 2014; thus, a minority of the children was 12–14 years of age at completion. The unique individual personal identification number assigned to all persons with a permanent residence in Denmark allowed a complete linkage on individual level between DNBC data and Danish nationwide registries containing comprehensive information on individual social characteristics and furthermore linkage between children and their parents [51]. We excluded participants with no information on spinal pain variables (*n* = 2848), maternal education (*n* = 47), equivalised household income (*n* = 182), siblings (*n* = 4), and family type (*n* = 153) (Fig. 1).

Data were stored and processed at Statistics Denmark and no personally identifiable data were accessible. Approval of the study was obtained from the Danish Data Protection Agency through the joint notification of the Faculty of Health and Medical Sciences at the University of Copenhagen and the DNBC Steering Committee.

### Information on spinal pain

**Fig. 2:**
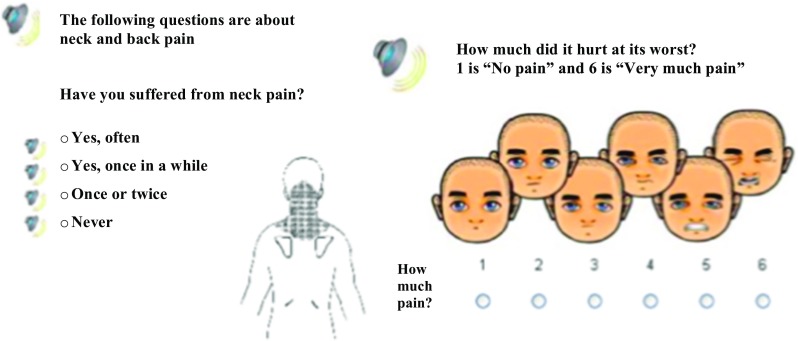
Illustration of original question for neck pain (frequency and intensity) included in DNBC-11. Identical questions were asked for middle back pain and low back pain. Rating of pain intensity was based on the Faces Pain Scale-Revised originally validated among 5–12-year-olds

DNBC-11 included a sub-division of the Young Spine Questionnaire (YSQ) (nine out of 19 questions), designed as a standardized tool of measuring spinal pain in children age 9–11 [32]. YSQ includes questions on pain frequency (often/once in a while/once or twice/never), pain intensity (1 “no pain” to 6 “very much pain”) of neck, middle back, and low back pain (Fig. 2), and a variety of daily-life consequences due to spinal pain [18, 32].

**Fig. 3:**
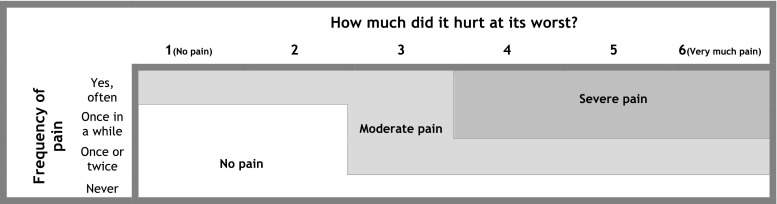
Illustration of composition of frequency and pain intensity for neck, middle back, and low back pain as well as the main outcome definition *overall spinal pain*

To distinguish between trivial and non-trivial pain [1, 16], we combined pain frequency and intensity for each spinal region into no pain, moderate pain, or severe pain. The optimal cut-point for consequential spinal pain in children is presently unknown, but based on findings from a previous study of children in this age group [1], also using the YSQ, severe pain was defined as pain of four or more on the Faces Pain Scale-Revised [18] and occurring at least “once in a while.” This definition has been used before in analyses of the present data [28]. Exact classification of pain groups appears from Fig. 3. Subsequently, we constructed the main outcome of interest *overall spinal pain* as a composite variable including the three spinal regions. If the pain reported differed between the three spinal locations, the location with the most severe pain was used (Fig. 3).

Children were considered to have *multiple spinal pain* if they reported severe pain in two or three spinal regions, and to have one-sited pain if they reported severe pain in one spinal region. *Spinal pain-related daily-life consequences* were a composite measure reflecting the number of daily-life consequences based on questions related to school absenteeism, physical activity restrictions, and healthcare utilization.

A variety of additional case-definitions of spinal pain were generated and applied in sensitivity analyses.

### Socio-demographic factors

A priori, we selected child’s age, sex, and additional socio-demographic factors as potential risk factors for spinal pain. Biological full siblings (having biological full siblings or not) and family type (living with both parents or not) were derived upon questions from DNBC-11. Maternal age at childbirth (≤ 25, 26–30, 31–35, > 35 years) was obtained from the Danish Medical Birth Registry [5]. Information on maternal education was obtained from the Danish Population’s Education Register [25]. Educational level was operationalized as the highest completed education attained the year of the child’s 11th birthday and was categorized into three groups according to the International Standard Classification of Education (ISCED) 2011: low (ISCED 0–2), medium (ISCED 3–4), and high (ISCED 5–8) [22]. Equivalised disposable income at the child’s 11th birthday was based on disposable household income extracted from the Income Statistics Register [3]. To enable comparison of family income across family size and composition, we divided disposable household income by an equivalence factor corresponding to the modified OECD scale. This method is available on OECD’s website. Equivalised disposable income was further categorized into quartiles by year relative to all mothers giving birth in the given year. All registries applied were available at Statistics Denmark.

### Statistical analyses

Descriptive statistics were reported using proportions and analyzed for heterogeneity using the chi-squared test. To examine associations between socio-demographic factors and different aspects of spinal pain, we applied crude and adjusted multinomial logistic regression models to estimate and report relative risk ratio (RRR) and their corresponding 95% confidence intervals (CI) [20] (for interpretation see Supplementary File 1). In all analyses, children with no pain were considered the reference outcome. The possibility of applying ordinal logistic regression models was investigated, but the proportional odds assumptions were not fulfilled [2].

To explore possible sex differences, we evaluated first-order interactions with child’s age and additional socio-demographic factors using a likelihood ratio test. The test showed no signs of interaction with the familial and socioeconomic factors; however, a statistically significant interaction was found between child’s sex and age. Hence, the regression analyses were adjusted for this interaction as well as for the main effects of the familial and socioeconomic factors. The dependency between siblings in the sample (*n* = 6416) was taken into account by applying a robust standard error estimator [53].

To account for sample selection (into the cohort and attrition) [23], we applied IPW in sub-analyses using all children born in Denmark from 1996 to 2003 as reference population (*n* = 505,690) [43]. The probability of participating in the study was estimated for each individual using the logistic regression model. For this purpose, we applied a given set of predictor variables for participation in DNBC-11. These factors included maternal education at childbirth, equivalised household income the year before birth, maternal parity, and urbanization, all obtained from Statistics Denmark and therefore available for participants as well as non-participants. Subsequently, the weight (i.e., the inverse of the probability of selection) was computed and included in the models. Thus, intuitively, each participant accounted for him/herself as well as for non-participants with similar characteristics [35].

The primary analyses were performed as complete case analyses and, subsequently, we performed a sensitivity analysis in which we accounted for missingness in DNBC-11 by applying multiple imputations on missing data for the included risk factors: family type, maternal education, and equivalised household income. The results remained essentially unchanged; hence, imputation was not applied in the study [38]. Finally, we conducted several additional sensitivity analyses to examine the robustness of the results.

All statistical analyses were performed using STATA V.15.

## Results

### Characteristics of children with spinal pain

Neck pain was the most frequent spinal region in which both girls and boys reported pain. Low back pain was the least frequent, but estimates were close to those of middle back pain (Table 1). Table 2 shows the composite definitions of spinal pain used to estimate the prevalence among children in DNBC-11 and the weighted prevalence relative to Danish children born from 1996 to 2003. In total, severe spinal pain (intensity 4–6) was reported in 9.8% of boys and 14.0% of girls, and moderate spinal pain in approximately 30% of all children. Intriguingly, the prevalence of spinal pain varied widely according to the applied case-definition of spinal pain. Including intensity 3 in the definition of severe spinal pain, the prevalence almost doubled, whereas an intensity of 5–6 reduced the estimates by half compared with the main outcome definition (data not shown). Most of the children only reported severe spinal pain in one spinal region. Approximately 23% of girls and 20% of boys had experienced at least one daily-life consequence due to spinal pain (Table 2) of which most cases were attributed to refrainment of physical activity (Table 1).

**Table 1:** Distribution of variables related to spinal pain available in the 11-year follow-up of the Danish National Birth Cohort, stratified by child’s sex (*N* = 46,726)

	Total *N* (%)	Boys *N* (%)	Girls *N* (%)
Neck pain^a^			
No pain	31,837 (68.1)	15,692 (70.4)	16,145 (66.0)
Moderate pain	11,399 (24.4)	5174 (23.2)	6225 (25.5)
Severe pain	3490 (7.5)	1421 (6.4)	2069 (8.5)
Middle back pain^a^			
No pain	38,354 (82.1)	18,640 (83.6)	19,714 (80.7)
Moderate pain	6399 (13.7)	2906 (13.1)	3493 (14.3)
Severe pain	1973 (4.2)	411 (3.3)	1232 (5.0)
Low back pain^a^			
No pain	40,154 (86.0)	19,769 (88.7)	20,385 (83.4)
Moderate pain	4847 (10.4)	1949 (8.8)	2898 (11.9)
Severe pain	1725 (3.7)	569 (2.6)	1156 (4.8)
School absenteeism^b^			
Never	43,476 (93.0)	20,892 (93.7)	22,584 (92.4)
1–2 times	2752 (5.9)	1180 (5.3)	1572 (6.5)
More than 2 times	487 (1.0)	209 (0.9)	278 (1.1)
Missing	11 (0.02)	6 (0.03)	5 (0.02)
Refrainment of physical activity^b^			
Never	39,809 (85.2)	19,181 (86.1)	20,628 (84.4)
1–2 times	5668 (12.1)	2604 (11.7)	3064 (12.5)
More than 2 times	1237 (2.7)	496 (2.2)	741 (3.0)
Missing	12 (0.03)	6 (0.03)	6 (0.02)
Care-seeking behavior^b^			
Never	42,262 (90.5)	20,311 (91.2)	21,951 (89.8)
1–2 times	2911 (6.2)	1346 (6.0)	1565 (6.4)
More than 2 times	1541 (3.3)	624 (2.8)	917 (3.8)
Missing	12 (0.03)	6 (0.03)	6 (0.02)

^a^Composite measure of pain frequency and intensity (see Figs. 2 and 3 for details)

^b^All variables are due to spinal pain and only available for children reporting pain in at least one spinal region

**Table 2:** Prevalence of spinal pain under different case-definitions among 46,726 Danish children from the Danish National Birth Cohort, 11–14 years of age, born between 1996 and 2003, stratified by child’s sex (chi-squared tests of heterogeneity between boys and girls were statistically significant for all case-definitions) (*N* = 46,726)

Case-definitions of spinal pain	Original data (unweighted data)			Weighted data^a^		
	Total *N* (%)	Boys *N* (%)	Girls *N* (%)	Total (%)	Boys (%)	Girls (%)
Overall spinal pain^b^						
No pain	27,256 (58.3)	13,667 (61.3)	13,589 (55.6)	57.3	60.1	54.7
Moderate pain	13,877 (29.7)	6446 (28.9)	7431 (30.4)	30.0	29.4	30.4
Severe pain	5593 (12.0)	2174 (9.8)	3419 (14.0)	12.8	10.4	14.9
Multiple spinal pain						
No pain	41,133 (88.0)	20,113 (90.2)	21,020 (86.0)	87.2	89.6	85.1
One-sited pain	4266 (9.1)	1705 (7.7)	2561 (10.5)	9.7	8.0	11.2
Multi-sited pain	1327 (2.9)	469 (2.1)	858 (3.5)	3.1	2.4	3.7
Spinal pain-related daily-life consequences^c^						
Never	36,541 (78.2)	17,736 (79.6)	18,805 (77.0)	77.6	79.1	76.2
1–2 times	7561 (16.2)	3471 (15.6)	4090 (16.8)	16.4	15.8	17.0
More than 2 times	2613 (5.6)	1074 (4.8)	1539 (6.3)	6.0	5.0	6.9
Missing	11 (0.02)	6 (0.03)	5 (0.02)	0.02	0.03	0.02

^a^Inverse probability weights relative to all children born in Denmark from 1996 to 2003

^b^Main outcome of interest based on neck, middle back, and low back pain (see Fig. 3)

^c^Defined as follows: “Never” if no experience of any of the daily-life consequences, “1–2 times” if they responded “once or twice” to only one of the daily-life consequences, and the remaining were categorized as “More than 2 times”

All selected socio-demographic factors were related to spinal pain (Table 3). Our findings indicated moderate and severe spinal pain to be more frequent among girls and the prevalence to increase rapidly with increasing age. Additionally, children with no biological siblings or children not living with both parents more often reported moderate and severe spinal pain. Likewise, children in less-educated and lower-income families were more likely to report severe spinal pain compared with those in high-status families. Similar patterns were observed for neck, middle back, and low back pain, separately (Supplementary File 2).

**Table 3:** Distribution of spinal pain according to selected characteristics of the 46,726 children participating in the 11-year follow-up in the Danish National Birth Cohort

	*N*	Overall spinal pain^ab^		
		No pain (%)	Moderate pain (%)	Severe pain (%)
Total population	46,726	58.3	29.7	12.0
Sex				
Boys	22,287	61.3	28.9	9.8
Girls	24,439	55.6	30.4	14.0
Age				
11 years	38,303	59.4	29.3	11.3
12 years	7226	54.0	31.3	14.7
13–14 years^c^	1197	49.7	32.9	17.6
Sibling position				
Biological full siblings	41,185	58.9	29.4	11.7
Only biological child	5541	53.9	31.7	14.4
Family type				
Child lives with both parents	36,533	59.9	29.1	11.0
Child not living with (both) parents^d^	10,193	52.7	32.0	15.3
Maternal educational level				
High	26,886	59.3	29.7	11.0
Medium	17,217	57.6	29.6	12.8
Low	2623	54.0	29.7	16.3
Equivalised household income				
4th quartile (highest)	16,698	60.3	28.9	10.9
3rd quartile	14,331	58.6	29.8	11.6
2nd quartile	10,542	57.0	30.3	12.7
1st quartile (lowest)	5,155	54.1	30.8	15.1
Maternal age at childbirth				
≤ 25 years	5417	55.6	30.8	13.6
26–30 years	19,972	58.4	29.6	12.0
31–35 years	16,043	58.7	29.7	12.0
> 35 years	5294	59.8	28.8	11.4

^a^Variables were analyzed with the chi-squared test of heterogeneity. Chi-squared tests were statistical significant for all variables

^b^For inverse probability weighted estimates see Supplementary File 3

^c^130 individuals were 14 years old, most of them just turned 14 years at time of follow-up

^d^Parents not living together due to divorce, separation, they never lived together, or only one parent alive

Applying IPW to account for selection, we observed a negligible increase in prevalence estimates, suggesting that results from DNBC may be applicable to estimate a population-based prevalence of spinal pain among children in Denmark (Table 2 and Supplementary File 3).

### The association between risk factors and spinal pain

The adjusted effect estimates of experiencing moderate or severe spinal pain confirmed the findings described above (Table 4). We observed, however, no clear association for maternal age at childbirth. Generally, the associations were stronger for severe pain than for moderate pain. In analyses of multiple spinal pain (i.e., pain in two or more regions) and daily-life consequences, we observed similar patterns to those of overall spinal pain (Table 5). The same applied when using alternative case-definitions (Supplementary Files 4–5). Despite small alterations, the overall findings on risk factors were unaffected by IPW (Supplementary Files 6–8).

**Table 4:** Relative risk ratio (RRR) of overall spinal pain according to potential risk factors among the 46,726 children participated in the 11-year follow-up in the Danish National Birth Cohort

Characteristics	Overall spinal pain			
	Model^ab^		Model 2^ac^	
	Moderate pain RRR (95% CI)	Severe pain RRR (95% CI)	Moderate pain RRR (95% CI)	Severe pain RRR (95% CI)
Sex, age				
Boys, 11 years	Ref.	Ref.	Ref.	Ref.
Boys, 12 years	1.12 (1.03–1.21)	1.16 (1.03–1.31)	1.12 (1.03–1.21)	1.15 (1.02–1.30)
Boys, 13+ years	1.41 (1.18–1.69)	1.29 (0.98–1.70)	1.41 (1.17–1.69)	1.26 (0.96–1.66)
Girls, 11 years	1.15 (1.10–1.20)	1.46 (1.37–1.56)	1.14 (1.09–1.19)	1.44 (1.35–1.54)
Girls, 12 years	1.41 (1.31–1.53)	2.39 (2.16–2.64)	1.41 (1.30–1.52)	2.34 (2.12–2.59)
Girls, 13+ years	1.49 (1.23–1.80)	3.47 (2.83–4.26)	1.48(1.23–1.79)	3.40 (2.77–4.18)
Sibling position				
Biological full siblings	Ref.	Ref.	Ref.	Ref.
Biological only child	1.18 (1.10–1.25)	1.35 (1.24–1.47)	1.12 (1.05–1.20)	1.18 (1.08–1.30)
Family type				
Child lives with both parents	Ref.	Ref.	Ref.	Ref.
Child not living with (both) parents^d^	1.25 (1.19–1.31)	1.57 (1.47–1.68)	1.18 (1.12–1.25)	1.39 (1.29–1.50)
Maternal educational level				
High	Ref.	Ref.	Ref.	Ref.
Medium	1.02 (0.98–1.07)	1.18 (1.13–1.27)	0.99 (0.94–1.03)	1.11 (1.05–1.19)
Low	1.08 (1.00–1.20)	1.62 (1.45–1.82)	1.01 (0.92–1.11)	1.37 (1.21–1.55)
Equivalised household income				
4th quartile (highest)	Ref.	Ref.	Ref.	Ref.
3rd quartile	1.06 (1.01–1.12)	1.10 (1.02–1.18)	1.05 (1.00–1.11)	1.06 (0.98–1.14)
2nd quartile	1.11 (1.05–1.17)	1.23 (1.14–1.33)	1.07 (1.01–1.13)	1.09 (1.00–1.18)
1st quartile (lowest)	1.19 (1.10–1.27)	1.55 (1.46–1.70)	1.10 (1.02–1.18)	1.23 (1.11–1.36)
Maternal age at childbirth				
≤ 25 years	Ref.	Ref.	Ref.	Ref.
26–30 years	0.92 (0.86–0.98)	0.84 (0.77–0.92)	0.96 (0.89–1.02)	0.95 (0.86–1.04)
31–35 years	0.91 (0.85–0.98)	0.81 (0.74–0.89)	0.95 (0.89–1.02)	0.91 (0.83–1.01)
> 35 years	0.87 (0.80–0.95)	0.78 (0.69–0.88)	0.90 (0.83–0.98)	0.87 (0.77–0.98)

^a^Reference category: not having reported moderate or severe spinal pain (no pain)

^b^Crude model

^c^Adjusted for additional variables in the model, as well as the interaction between child’s age and sex (*P* < 0.001)

^d^Parents not living together due to divorce, separation, they never lived together, or only one parent alive

**Table 5:** Relative risk ratio (RRR) of multiple spinal pain and daily-life consequences due to spinal pain, respectively, according to potential risk factors among the 46,726 children participated in the 11-year follow-up in the Danish National Birth Cohort

Characteristics	No. of cases (One-sited/Multi-sited)	Multiple spinal pain^ac^		No. of cases (1–2 times/More than two times)	Daily-life consequences^bc^	
		One-sited RRR (95% CI)	Multi-sited RRR (95% CI)		1–2 times RRR (95% CI)	More than two times RRR (95% CI)
Sex, age						
Boys, 11 years	1377/376	Ref.	Ref.	2714/863	Ref.	Ref.
Boys, 12 years	283/75	1.12 (0.98–1.28)	1.08 (0.84–1.39)	639/189	1.36 (1.23–1.50)	1.26 (1.07–1.48)
Boys, 13+ years	45/18	1.02 (0.75–1.39)	1.47 (0.91–2.38)	118/22	1.44 (1.17–1.78)	0.83 (0.54–1.27)
Girls, 11 years	1963/606	1.34 (1.25–1.45)	1.51 (1.33–1.73)	3176/1167	1.10 (1.04–1.16)	1.26 (1.15–1.39)
Girls, 12 years	496/206	1.87 (1.67–2.09)	2.81 (2.36–3.35)	779/303	1.53 (1.40–1.67)	1.84 (1.60–2.11)
Girls, 13+ years	102/46	2.60 (2.08–3.24)	4.21 (3.05-5.82)	135/69	1.79 (1.47–2.18)	2.81 (2.15–3.68)
Sibling position						
Biological full siblings	3656/1140	Ref.	Ref.	6598/2221	Ref.	Ref.
Biological only child	610/187	1.15 (1.04–1.26)	1.10 (0.93–1.31)	963/392	1.08 (1.00–1.17)	1.24 (1.10–1.40)
Family type						
Child lives with both parents	3087/948	Ref.	Ref.	5709/1918	Ref.	Ref.
Child not living with (both) parents^d^	1179/379	1.31 (1.21–1.42)	1.32 (1.15–1.51)	1852/695	1.17 (1.10–1.25)	1.20 (1.09–1.33)
Maternal educational level						
High	2308/652	Ref.	Ref.	4415/1364	Ref.	Ref.
Medium	1636/570	1.07 (1.00–1.14)	1.30 (1.15–1.46)	2708/1035	0.92 (0.87–0.97)	1.13 (1.03–1.23)
Low	322/105	1.33 (1.17–1.52)	1.47 (1.18–1.84)	438/214	0.96 (0.86–1.08)	1.47 (1.25–1.72)
Equivalised household income						
4th quartile (highest)	1398/416	Ref.	Ref.	2566/884	Ref.	Ref.
3rd quartile	1281/382	1.04 (0.96–1.13)	1.03 (0.89–1.18)	2357/736	1.09 (1.02–1.16)	0.96 (0.86–1.06)
2nd quartile	1015/321	1.06 (0.97–1.16)	1.08 (0.93–1.26)	1738/616	1.07 (1.00–1.15)	1.03 (0.92–1.15)
1st quartile (lowest)	572/208	1.15 (1.02–1.28)	1.32 (1.10–1.)	900/377	1.13 (1.03–1.24)	1.22 (1.06–1.40)
Maternal age at childbirth						
≤ 25 years	565/170	Ref.	Ref.	945/320	Ref.	Ref.
26–30 years	1833/564	0.95 (0.85–1.05)	1.01 (0.85–1.21)	3205/1107	0.93 (0.86–1.01)	1.02 (0.89–1.16)
31–35 years	1408/451	0.90 (0.81–1.00)	1.01 (0.84–1.22)	2560/890	0.92 (0.85–1.00)	1.02 (0.89–1.16)
> 35 years	460/142	0.88 (0.77–1.00)	0.97 (0.76–1.22)	851/296	0.93 (0.84–1.03)	1.00 (0.85–1.19)

^a^Reference category: not having reported in any of the spinal regions

^b^Reference category: not having experienced daily-life consequences due to spinal pain

^c^Adjusted for additional variables in the model, as well as the interaction between child’s age and sex (*P* < 0.001)

^d^Parents not living together due to divorce, separation, they never lived together, or only one parent alive

In sensitivity analyses, we examined the robustness of the associations using maternal education at childbirth and household income the year before birth instead of at the year of the child’s 11th birthday, for which the same effect estimates were observed. The same applied when using parental education (i.e., the highest attained education of the parents) instead of maternal education.

## Discussion

In this descriptive paper using data from 46,726 children in DNBC, we demonstrated that a sizeable number of children aged 11 to 14 suffered from moderate or severe spinal pain. Spinal pain was more common in girls and increased with age. Further, the results displayed a clear social gradient in the experience of spinal pain. The findings were confirmed after IPW, taking selection into account.

The results emphasize spinal pain to be a common problem among children; however, the exact extent of the problem depends highly on the case-definition and nature of spinal pain as well as the age of study participants. We found prevalence estimates of severe spinal pain in 11–14 year-olds to be almost 10% for boys and 14% for girls with a rapid increase with increasing age. Previous studies included individuals up to age 23 [27] (i.e., allowing the prevalence to increase with age), and thereby complicating comparison of estimates. The differential nature of spinal pain implies a need for careful application of case-definitions, interpretation of results, and in the planning of preventive strategies. Studies have suggested that having pain often or once in a while can be interpreted as an indicator of recurrent pain, which may cause marked discomfort and impairment in children’s everyday life and reduce their quality of life as well as causing lifelong problems with pain [42, 46]. Persistent pain has been associated with the co-occurrence of other symptoms, physically and psychological [44], and in a Danish twin study adolescents with persistent low back pain were 3.5 times more likely to have persistent low back pain as adults [16]. Alongside, co-occurrence of other musculoskeletal symptoms is hypothesized as a risk indicator for a more persistent course (i.e., multiple spinal pain) [44]. Oppositely, having experienced a single episode of pain can either be due to a sudden trauma or injury, or the beginning of a pain trajectory with insidious pain onset [9]. The latter is especially interesting for the age group included in our study, since this age has been suggested as spinal pain onset. We grouped infrequent pain with low intensity as no pain. It is possible that these children were in the beginning of a pain trajectory, thus, may belong to the group of children that may benefit from appropriate support to prevent spinal pain later in life.

Socioeconomic status is associated with parents’ ability to affect their children’s health and well-being in a positive manner due to lifestyle, health behavior, and knowledge [14]. Thus, children growing up in disadvantaged families are predisposed to health adversities [8, 41]. We found children from less-educated and lower-income families to be more likely to experience spinal pain compared with children in well-off families. These findings are in accordance with two Nordic studies suggesting a similar social gradient in somatic complaints, including back pain [13, 41], and with a recent systematic review suggesting low socioeconomic status to be a risk factor for onset of musculoskeletal pain in studies with long-term follow-up [21].

We also found children with no biological full siblings and children in separated families to be more likely to experience spinal pain. The family situation may affect the vulnerability and well-being of the child. In line with our findings, studies have shown that children in single-parent families, in stepfamilies, or only children were more vulnerable and had worse health outcomes than children in traditional families or children with siblings, and further that health adversities hereof psychosomatic symptoms were more common among these children [41, 52]. When familial and socioeconomic variables were introduced in the models, the effect estimates were only slightly reduced and remained statistically significant, indicating that familial determinants were still affecting childhood spinal pain when adjusting for socioeconomic factors, and vice versa. Thus, it is likely that some of the underlying mechanisms may be found within the family environment of the child (i.e., affecting vulnerability and well-being of the child) such as in parental pain behavior [7, 47], chronic pain, parental mental health and behavioral problems (i.e., depression, anxiety, and substance use) [19, 40], or in other psychosocial and lifestyle factors [12, 26]. These conditions might impact psychological symptoms in the child such as sleep difficulties, feeling low, nervousness, general well-being, and loneliness which have previously been associated with spinal pain in children [4, 45].

### Strengths and limitations

As one of the few, DNBC facilitates large-scale life-course studies of spinal pain etiology and prevention due to the great inclusion of validated self-reported spinal pain questions on more than 46,000 11–14-year-olds as well as rich data on exposures from conception and onwards, i.e., potential familial risk factors for spinal pain. Since DNBC is nested within the Danish population, it allows individual linkage of data on health and (parental) social issues from Danish nationwide registries, permitting analyses of, e.g., any social interactions in the disease production. For this study, linkage to Danish registries made it possible to provide a population-based estimate of spinal pain prevalence using inverse probability weights relative to all children born in Denmark from 1996 to 2003.

Some limitations of the study are worth mentioning to ensure accurate interpretation of the results. Generally, the cross-sectional design impedes causal conclusions. However, sensitivity analyses on risk factors occurring before spinal pain onset as well as knowledge upon spinal pain onset to occur around age 10–12 [6, 9, 24, 31, 33] strengthen the study temporality.

In DNBC-11, information on spinal pain was based on children’s self-report. Children’s perception of pain is subjective and self-reported data are prone to induce misclassification; however, children’s self-report has previously been defined as a reliable approach to measuring pain in children [50]. It should, however, be taken into account that the child’s vulnerability, health and well-being, age, sex, cognitive level, and familial background may affect their pain reports [34, 50].

Among the children that participated in DNBC-11, 5.7% were excluded due to incomplete data on spinal pain variables. Imputation of an outcome measure is inadequate, and further, we cannot rule out that data are missing not at random; thus, the estimates may be biased [38]. Nonetheless, applying IPW is a method to reduce bias from complete case analyses [43].

DNBC participants are a selected sample of the source population with participation strongly related to familial and socioeconomic factors [23]. When accounting for sample selection by applying IPW [35, 43], we found the impact on the estimates to be negligible. This is in accordance with methodological findings by Jacobsen et al. and Pizzi et al. investigating the impact of selection in birth cohort studies [23, 39]. However, since IPW does not address unknown or unmeasured factors that influence selection, fully representative estimates for the Danish population cannot be concluded [35]. Despite potential selection problems, the advantages of using detailed birth cohort data should be balanced against issues of study validity, selection, and being the only possible approach to perform large-scale life-course studies on childhood spinal pain.

## Conclusion

A considerable number of children suffer from spinal pain. Spinal pain is more common in girls and the prevalence increases with increasing age. In addition, children in more disadvantaged families are more likely to experience spinal pain. Awareness of the consequences of applying different case-definitions is essential in the assessment of spinal pain. Our findings provide a basis for further in-depth examination of spinal pain etiology with the aim of informing efficient and targeted prevention of spinal pain.

## Electronic supplementary material

ESM 1 (PDF 441 kb)

## Abbreviations

DNBC: The Danish National Birth Cohort
DNBC-11: The 11-year follow-up in the Danish National Birth Cohort
IPW: Inverse probability weighting
ISCED: International Standard Classification of Education
RRR: Relative risk ratio
Spinal pain: Neck pain, middle back pain, and/or low back pain
YSQ: The Young Spine Questionnaire
95% CI: 95% confidence intervals
