# Supplementary material for: Epidemiology of spinal pain in children: a study within the Danish National Birth Cohort
Source: Eur J Pediatr. 2019 Feb 20;178(5):695–706. doi: 10.1007/s00431-019-03326-7 (PMC6459805; doi:10.1007/s00431-019-03326-7)
Supplement: Supplementary file 1 — (PDF 441 kb) [file 431_2019_3326_MOESM1_ESM.pdf]

SUPPLEMENTARY FILES TO PAPER:

Title: Epidemiology of spinal pain in children: a study within The Danish National Birth Cohort

Journal: European Journal of Pediatrics

Authors:
Anne Cathrine Joergensen[1], Lise Hestbaek[2], Per Kragh Andersen[3], Anne-Marie Nybo Andersen[1]

Affiliation: [1] Section of Epidemiology, Department of Public Health, Faculty of Health and Medical Science, University of Copenhagen, Oster Farimagsgade 5, bd. 24, DK-1014 Copenhagen K, Denmark Denmark
[2] Department of Sport Science and Clinical Biomechanics, University of Southern Denmark, Campusvej 55, 5230 Odense, Denmark
[3] Section of Biostatistics, Department of Public Health, Faculty of Health and Medical Science, University of Copenhagen, Oster Farimagsgade 5, 1014 Copenhagen K, Denmark

Address corresponding to: Anne Cathrine Joergensen, Section of Epidemiology, Department of Public Health, Faculty of Health and Medical Science, University of Copenhagen, Oster Farimagsgade 5, bd. 24, Box 2099, DK-1014 Copenhagen K, Denmark, e-mail: acjo@sund.ku.dk, +4561776975

Supplementary File 1: Interpretation of relative risk ratio in multinomial logistic regression models

Children with no pain (Y=0) were considered as the reference outcome in the analyses.

For a binary covariate (Z=1 vs. Z=0) the regression coefficient b_j for a given level (Y=j) of spinal pain can then be interpreted via a relative risk ratio (RRR).

Thus, exp(b_j)=(P(Y=j|Z=1)/P(Y=j|Z=0))/(P(Y=0|Z=1)/P(Y=0|Z=0)), i.e. the risk ratio for the outcome Y=j compared to that of Y=0.

Supplementary File 2

Distribution of variables related to spinal pain available in the 11-year follow-up of The Danish National Birth Cohort, according to specific spinal region[a] and stratified by child's sex. (N = 46,726)

	Boys						Girls					
	**Neck pain**		**Middle back pain**		**Low back pain**		**Neck pain**		**Middle back pain**		**Low back pain**	
	No	Yes	No	Yes	No	Yes	No	Yes	No	Yes	No	Yes
Characteristics	20,866 (93.6)	1,421 (6.4)	21,546 (96.7)	741 (3.3)	21,718 (97.5)	569 (2.6)	22,370 (91.5)	2,069 (8.5)	23,207 (95.0)	1,232 (5.0)	23,283 (95.3)	1,156 (4.7)
Age												
11 years	17,150 (82.2)	1,172 (82.5)	17,719 (82.2)	603 (81.4)	17,898 (82.4)	424 (74.5)	18,360 (82.1)	1,621 (78.4)	19,078 (82.2)	903 (73.3)	19,210 (82.5)	771 (66.7)
12 years	3,160 (15.1)	221 (15.6)	3,272 (15.2)	109 (14.7)	3,260 (15.0)	121 (21.3)	3,482 (15.6)	363 (17.5)	3,572 (15.4)	273 (22.2)	3,527 (15.2)	318 (27.5)
13 + years	556 (2.7)	28 (2.0)	555 (2.6)	29 (3.9)	560 (2.6)	24 (4.2)	528 (2.4)	85 (4.1)	557 (2.4)	56 (4.6)	546 (2.4)	67 (5.8)
Sibling position												
Biological full siblings	18,422 (88.3)	1,229 (86.5)	19,007 (88.2)	644 (86.9)	19,176 (88.3)	475 (83.5)	19,767 (88.4)	1,767 (85.4)	20,468 (88.6)	1,066 (86.5)	20,557 (88.3)	977 (84.5)
Biological only child	2,444 (11.7)	192 (13.5)	2,539 (11.8)	97 (13.1)	2,542 (11.7)	94 (16.5)	2,603 (11.6)	302 (14.6)	2,739 (11.8)	166 (13.5)	2,726 (11.7)	179 (15.5)
Family type[b]												
Child lives with both parents	16,632 (80.0)	1,050 (74.3)	17,146 (79.9)	536 (72.5)	17,262 (79.8)	420 (74.1)	17,381 (78.1)	1,470 (72.0)	17,987 (77.9)	864 (71.1)	18,025 (77.8)	826 (72.1)
Child not living with (both) parents[b]	4,160 (20.0)	364 (25.7)	4,321 (20.1)	203 (27.5)	4,377 (20.2)	147 (26.0)	4,880 (21.9)	572 (28.0)	5,101 (22.1)	351 (28.9)	5,132 (22.2)	320 (27.8)
Maternal educational level												
High	12,263 (58.8)	762 (53.6)	12,640 (58.7)	385 (52.0)	12,716 (58.6)	309 (54.3)	12,801 (57.2)	1,060 (51.2)	13,243 (57.1)	618 (50.2)	13,258 (57.0)	603 (52.2)
Medium	7,487 (35.9)	547 (38.5)	7,751 (36.0)	283 (38.2)	7,811 (36.0)	223 (39.2)	8,330 (37.2)	853 (41.2)	8.660 (37.3)	523 (42.5)	8,717 (37.4)	466 (40.3)
Low	1,116 (5.4)	112 (7.9)	1,155 (5.4)	73 (9.9)	1,191 (5.5)	37 (6.5)	1,239 (5.5)	156 (7.5)	1,304 (5.6)	91 (7.4)	1,308 (5.6)	87 (7.5)
Equivalised household income												
4th quartile (highest)	7,689 (36.9)	442 (31.1)	7,890 (36.6)	241 (32.5)	7,951 (36.6)	180 (31.6)	7,904 (35.3)	663 (32.0)	8,184 (35.3)	383 (31.1)	8,167 (35.1)	400 (34.6)
3rd quartile	6,431 (30.8)	425 (29.9)	6,663 (30.9)	193 (26.1)	6,682 (30.8)	174 (30.6)	6,859 (30.7)	616 (29.8)	7,117 (30.7)	358 (29.1)	7,129 (30.6)	346 (29.9)
2nd quartile	4,594 (22.0)	356 (25.1)	4,755 (22.1)	195 (26.3)	4,805 (22.1)	145 (25.5)	5,117 (22.9)	475 (23.0)	5,291 (22.8)	301 (24.4)	5,331 (22.9)	261 (22.6)
1st quartile (lowest)	2,152 (10.3)	198 (13.9)	2,238 (10.4)	112 (15.1)	2,280 (10.5)	70 (12.3)	2,490 (11.1)	315 (15.2)	2,615 (11.3)	190 (15.4)	2,656 (11.4)	149 (12.9)
Maternal age at childbirth												
≤ 25 years	2,377 (11.4)	187 (13.2)	2,469 (11.5)	95 (12.8)	2,486 (11.5)	78 (13.7)	2,571 (11.5)	282 (13.6)	2,700 (11.6)	153 (12.4)	2,708 (11.6)	145 (12.5)
26-30 years	8,890 (42.6)	609 (42.9)	9,190 (42.7)	309 (41.7)	9,253 (42.6)	246 (43.2)	9,595 (42.9)	878 (42.4)	9,937 (42.8)	536 (43.5)	9,974 (42.8)	499 (43.2)
31-35 years	7,182 (34.4)	480 (33.8)	7,409 (34.4)	253 (34.1)	7,477 (34.4)	185 (32.5)	7,694 (34.4)	687 (33.2)	7,960 (34.3)	421 (34.2)	8,008 (34.4)	373 (32.3)
> 35 years	2,417 (11.6)	145 (10.2)	2,478 (11.5)	84 (11.3)	2,502 (11.5)	60 (10.5)	2,510 (11.2)	222 (10.7)	2,610 (11.3)	122 (9.9)	2,593 (11.1)	139 (12.0)

a Binary variables; Yes: Similar to severe pain. No: No pain and moderate pain lumped together

b Parents not living together due to divorce, separation, they never lived together or only one parent alive.

Supplementary File 3

Weighted distribution of spinal pain according to selected characteristics using inverse probability weights relative to all children born in Denmark from 1996-2003.

	No pain (%)	Moderate pain (%)	Severe pain (%)
Sex			
Boys	60.1	29.4	10.4
Girls	54.7	30.4	14.9
Age			
11 years	58.4	29.6	12.0
12 years	53.6	30.9	15.5
13 + years	47.6	33.0	19.5
Sibling position			
Biological full siblings	57.9	29.7	12.4
Only biological child	53.5	31.4	15.1
Family type			
Child lives with both parents	59.2	29.1	11.8
Child not living with (both) parents	51.9	32.4	15.7
Maternal educational level			
High	58.7	30.1	11.2
Medium	56.9	30.0	13.1
Low	54.4	29.3	16.4
Equivalised household income			
4th quartile (highest)	60.1	28.8	11.2
3rd quartile	57.8	30.2	12.0
2nd quartile	56.7	30.1	13.3
1st quartile (lowest)	52.2	31.4	16.4
Maternal age at childbirth			
≤ 25 years	53.8	31.8	14.4
26-30 years	57.6	29.8	12.6
31-35 years	57.9	29.6	12.5
> 35 years	58.9	28.9	12.2

Supplementary File 4

Relative risk ratio (RRR) for spinal pain (severe spinal pain intensity 5-6) according to potential risk factors among the 46,726 children participated in the 11-year follow-up in The Danish National Birth Cohort

	Moderate pain[bc]<br>RRR (95% CI)	Severe pain[abc]<br>RRR (95% CI)
Sex, age		
Boys, 11 years	Ref.	Ref.
Boys, 12 years	1.14 (1.06-1.23)	0.95 (0.77-1.17)
Boys, 13+ years	1.40 (1.18-1.66)	1.10 (0.70-1.74)
Girls, 11 years	1.18 (1.13-1.23)	1.58 (1.43-1.75)
Girls, 12 years	1.53 (1.42-1.65)	2.76 (2.38-3.19)
Girls, 13+ years	1.77 (1.49-2.09)	3.97 (2.97-5.29)
Sibling position		
Biological full siblings	Ref.	Ref.
Biological only child	1.13 (1.06-1.20)	1.25 (1.09-1.43)
Family type		
Child lives with both parents	Ref.	Ref.
Child not living with (both) parents[d]	1.22 (1.16-1.29)	1.40 (1.26-1.57)
Maternal educational level		
High	Ref.	Ref.
Medium	1.00 (0.96-1.05)	1.18 (1.07-1.29)
Low	1.07 (0.98-1.17)	1.41 (1.17-1.69)
Equivalised household income		
4th quartile (highest)	Ref.	Ref.
3rd quartile	1.05 (1.00-1.11)	1.05 (0.94-1.19)
2nd quartile	1.06 (1.01-1.12)	1.14 (1.01-1.29)
1st quartile (lowest)	1.11 (1.03-1.19)	1.36 (1.17-1.58)
Maternal age at childbirth		
≤ 25 years	Ref.	Ref.
26-30 years	0.95 (0.89-1.01)	0.99 (0.85-1.14)
31-35 years	0.94 (0.88-1.00)	0.95 (0.82-1.11)
> 35 years	0.89 (0.82-0.96)	0.93 (0.77-1.12)

a Severe spinal pain defined as pain of five or more on the Faces Pain Scale-Revised and occurring at least 'once in a while'

b Reference category: Not having reported moderate or severe spinal pain (No pain)

c Adjusted for additional variables in the model, as well as the interaction between child´s age and sex (P<0.001)

Supplementary File 5

Relative risk ratio (RRR) for spinal pain (severe spinal pain intensity 3-6) according to potential risk factors among the 46,726 children participated in the 11-year follow-up in The Danish National Birth Cohort

	Moderate pain[bc] RRR (95% CI)	Severe pain[abc] RRR (95% CI)
Sex, age		
Boys, 11 years	Ref.	Ref.
Boys, 12 years	1.06 (0.97-1.17)	1.20 (1.09-1.32)
Boys, 13+ years	1.34 (1.09-1.64)	1.41 (1.15-1.74)
Girls, 11 years	1.10 (1.04-1.15)	1.36 (1.29-1.44)
Girls, 12 years	1.18 (1.08-1.30)	2.20 (2.02-2.39)
Girls, 13+ years	1.32 (1.06-1.64)	2.74 (2.28-3.30)
Sibling position		
Biological full siblings	Ref.	Ref.
Biological only child	1.11 (1.03-1.20)	1.17 (1.08-1.26)
Family type		
Child lives with both parents	Ref.	Ref.
Child not living with (both) parents[d]	1.16 (1.09-1.23)	1.33 (1.25-1.41)
Maternal educational level (11 years)		
High	Ref.	Ref.
Medium	0.97 (0.92-1.02)	1.08 (1.03-1.14)
Low	0.95 (0.86-1.06)	1.27 (1.14-1.40)
Equivalised household income (11 years)		
4th quartile (highest)	Ref.	Ref.
3rd quartile	1.03 (0.98-1.10)	1.07 (1.01-1.14)
2nd quartile	1.04 (0.97-1.11)	1.11 (1.04-1.18)
1st quartile (lowest)	1.06 (0.97-1.16)	1.21 (1.11-1.31)
Maternal age at childbirth		
≤ 25 years	Ref.	Ref.
26-30 years	0.99 (0.91-1.06)	0.92 (0.86-1.00)
31-35 years	0.97 (0.90-1.06)	0.91 (0.84-0.99)
> 35 years	0.90 (0.81-1.00)	0.88 (0.80-0.98)

a Severe spinal pain defined as pain of three or more on the Faces Pain Scale-Revised and occurring at least ‘once in a while’

b Reference category: Not having reported moderate or severe spinal pain (No pain)

c Adjusted for additional variables in the model, as well as the interaction between child´s age and sex (P<0.001)

Supplementary File 6

Weighted estimates of relative risk ratio[ab] (RRR) for overall spinal pain using inverse probability weights relative to all children born in Denmark from 1996 to 2003.

	Moderate pain RRR (95% CI)	Severe pain RRR (95% CI)
Sex, age		
Boys, 11 years	Ref.	Ref.
Boys, 12 years	1.12 (1.02-1.24)	1.21 (1.04-1.40)
Boys, 13+ years	1.49 (1.19-1.86)	1.35 (0.98-1.86)
Girls, 11 years	1.13 (1.06-1.20)	1.45 (1.34-1.57)
Girls, 12 years	1.29 (1.13-1.47)	2.16 (1.85-2.50)
Girls, 13+ years	1.41 (1.12-1.76)	3.56 (2.71-4.66)
Sibling position		
Biological full siblings	Ref.	Ref.
Biological only child	1.08 (0.98-1.19)	1.15 (1.01-1.30)
Family type		
Child lives with both parents	Ref.	Ref.
Child not living with (both) parents[d]	1.20 (1.10-1.30)	1.30 (1.17-1.44)
Maternal educational level (11 years)		
High	Ref.	Ref.
Medium	0.99 (0.94-1.04)	1.11 (1.04-1.19)
Low	0.95 (0.82-1.10)	1.30 (1.10-1.53)
Equivalised household income (11 years)		
4th quartile (highest)	Ref.	Ref.
3rd quartile	1.08 (1.01-1.15)	1.08 (0.98-1.18)
2nd quartile	1.06 (0.98-1.15)	1.13 (1.01-1.26)
1st quartile (lowest)	1.16 (1.04-1.30)	1.35 (1.17-1.56)
Maternal age at childbirth		
≤ 25 years	Ref.	Ref.
26-30 years	0.92 (0.84-1.01)	0.92 (0.82-1.03)
31-35 years	0.91 (0.83-1.00)	0.91 (0.81-1.03)
> 35 years	0.87 (0.79-0.97)	0.86 (0.75-1.00)

a Reference category: Not having reported moderate or severe spinal pain (No pain)
b Adjusted for additional variables in the model, as well as the interaction between child´s age and sex (P<0.001)

Supplementary File 7

Weighted estimates of relative risk ratio[ab] (RRR) for multiple spinal pain using inverse probability weights relative to all children born in Denmark from 1996 to 2003.

	One-sited RRR (95% CI)	Multi-sited RRR (95% CI)
Sex, age		
Boys, 11 years	Ref.	Ref.
Boys, 12 years	1.18 (1.01-1.38)	1.10 (0.81-1.49)
Boys, 13+ years	1.07 (0.75-1.52)	1.50 (0.85-2.65)
Girls, 11 years	1.38 (1.26-1.50)	1.43 (1.23-1.67)
Girls, 12 years	1.81 (1.57-2.09)	2.48 (2.00-3.07)
Girls, 13+ years	2.97 (2.21-4.00)	3.70 (2.47-5.50)
Sibling position		
Biological full siblings	Ref.	Ref.
Biological only child	1.13 (1.00-1.29)	1.05 (0.85-1.29)
Family type		
Child lives with both parents	Ref.	Ref.
Child not living with (both) parents[d]	1.20 (1.08-1.33)	1.26 (1.07-1.48)
Maternal educational level		
High	Ref.	Ref.
Medium	1.07 (0.99-1.16)	1.26 (1.11-1.44)
Low	1.28 (1.10-1.50)	1.44 (1.13-1.83)
Equivalised household income		
4th quartile (highest)	Ref.	Ref.
3rd quartile	1.05 (0.96-1.16)	1.04 (0.88-1.23)
2nd quartile	1.11 (0.99-1.24)	1.09 (0.91-1.30)
1st quartile (lowest)	1.26 (1.09-1.45)	1.36 (1.09-1.69)
Maternal age at childbirth		
≤ 25 years	Ref.	Ref.
26-30 years	0.91 (0.81-1.03)	1.08 (0.88-1.32)
31-35 years	0.90 (0.79-1.02)	1.07 (0.87-1.33)
> 35 years	0.86 (0.74-1.01)	1.06 (0.81-1.40)

a Reference category: Not having reported severe spinal pain in any of the spinal regions

b Adjusted for additional variables in the model, as well as the interaction between child´s age and sex (P<0.001)

Supplementary File 8

Weighted estimates of relative risk ratio[ab] (RRR) for spinal pain-related daily-life consequences using inverse probability weights relative to all children born in Denmark from 1996 to 2003.

	1-2 times RRR (95% CI)	More than two times RRR (95% CI)
Sex, age		
Boys, 11 years	Ref.	Ref.
Boys, 12 years	1.35 (1.20-1.52)	1.22 (1.01-1.48)
Boys, 13+ years	1.38 (1.06-1.80)	1.05 (0.63-1.76)
Girls, 11 years	1.10 (1.02-1.19)	1.33 (1.20-1.49)
Girls, 12 years	1.45 (1.28-1.65)	1.82 (1.52-2.17)
Girls, 13+ years	1.94 (1.48-2.54)	2.67 (1.96-3.65)
Sibling position		
Biological full siblings	Ref.	Ref.
Biological only child	1.06 (0.96-1.18)	1.23 (1.05-1.43)
Family type		
Child lives with both parents	Ref.	Ref.
Child not living with (both) parents[d]	1.14 (1.05-1.24)	1.20 (1.06-1.37)
Maternal educational level		
High	Ref.	Ref.
Medium	0.93 (0.88-0.99)	1.13 (1.03-1.24)
Low	0.98 (0.83-1.16)	1.34 (1.11-1.60)
Equivalised household income		
4th quartile (highest)	Ref.	Ref.
3rd quartile	1.12 (1.03-1.21)	0.99 (0.88-1.12)
2nd quartile	1.08 (0.99-1.18)	1.11 (0.97-1.27)
1st quartile (lowest)	1.15 (1.02-1.29)	1.30 (1.10-1.53)
Maternal age at childbirth		
≤ 25 years	Ref.	Ref.
26-30 years	0.93 (0.84-1.02)	0.96 (0.83-1.12)
31-35 years	0.92 (0.84-1.02)	0.97 (0.83-1.13)
> 35 years	0.96 (0.84-1.09)	1.00 (0.82-1.23)

a Reference category: Not having reported any daily-life consequences due to spinal pain

b Adjusted for additional variables in the model, as well as the interaction between child´s age and sex (P<0.001)
